# An empirical study of race times in recreational endurance runners

**DOI:** 10.1186/s13102-016-0052-y

**Published:** 2016-08-26

**Authors:** Andrew J. Vickers, Emily A. Vertosick

**Affiliations:** Memorial Sloan Kettering Cancer Center, 485 Lexington Avenue, New York, NY 10017 USA

**Keywords:** Performance, Running, Sports training, Prediction modeling

## Abstract

**Background:**

Studies of endurance running have typically involved elite athletes, small sample sizes and measures that require special expertise or equipment.

**Methods:**

We examined factors associated with race performance and explored methods for race time prediction using information routinely available to a recreational runner. An Internet survey was used to collect data from recreational endurance runners (*N* = 2303). The cohort was split 2:1 into a training set and validation set to create models to predict race time.

**Results:**

Sex, age, BMI and race training were associated with mean race velocity for all race distances. The difference in velocity between males and females decreased with increasing distance. Tempo runs were more strongly associated with velocity for shorter distances, while typical weekly training mileage and interval training had similar associations with velocity for all race distances. The commonly used Riegel formula for race time prediction was well-calibrated for races up to a half-marathon, but dramatically underestimated marathon time, giving times at least 10 min too fast for half of runners. We built two models to predict marathon time. The mean squared error for Riegel was 381 compared to 228 (model based on one prior race) and 208 (model based on two prior races).

**Conclusions:**

Our findings can be used to inform race training and to provide more accurate race time predictions for better pacing.

**Electronic supplementary material:**

The online version of this article (doi:10.1186/s13102-016-0052-y) contains supplementary material, which is available to authorized users.

## Background

Many millions of recreational runners compete in long-distance races each year. One key question for such runners concerns factors associated with performance. Modifiable factors, such as training, may suggest changes that a runner might make to improve race times; factors that cannot be modified, such as age or sex, can be used to make fair comparisons between different runners. A second important question for long-distance runners concerns race time prediction, critical for pacing during the early stages of a race. Race time predictors are widely available on the Web, and typically predict time of a future race on the basis of previous race of a different distance [1]. For instance, a user might be asked to enter the time of a recent 10 km race in order to predict the time of a forthcoming marathon. Factors associated with race time and race time prediction are related but quite distinct scientific questions. It may be, for instance, that interval training is associated with race time, but does not help predict time for a longer race on the basis of time for a shorter race, because interval training improves velocity at both distances.

There are reasons to believe that both sets of questions – factors related to performance and race time prediction – have been poorly addressed for the recreational runner. The first problem is that much of the literature has focused on elite runners. For instance, several studies on the effects of sex on long-distance running performance have been based on world record times [2, 3]. A study assessing the relationship between training volume and marathon times was based on athletes who qualified for the US Olympic marathon trials [4]. In a an expansive literature review of the value of interval, threshold and “long slow distance training”, Seiler et al. evaluated numerous studies on elite athletes before asking whether the findings could be applied to recreational athletes, concluding that “there are almost no published data addressing this question” [5]. Most race time predictors are based on the Riegel formula, derived from a paper that analyzed world record times for a variety of endurance sports [6].

The recreational runner faces a second problem with the literature, which is that many studies report on measures that require special expertise or equipment. While these are of value for understanding mechanisms of training effects and for predicting race times, they are of less use for recreational runners who lack access to these tools. Typical studies have predicted race time based on the results of cycle ergometry [7], skinfold assessment of body fat [8], or ventilatory threshold determined from an incremental treadmill test [9]. Other studies are weakened by limited sample sizes. Studies on race time prediction have included sample sizes such as 84 [10], 29 [11], or in two cases, as low as 12 [9, 12].

We aimed to collect routinely available data from a large sample of recreational runners in order to understand factors associated with race performance, and to develop a prediction model to aid race pacing for the marathon and other races.

## Methods

### Experimental approaches to the problem

We explored factors associated with race performance and methods for race time prediction. One approach would have been to download databases of race times from large races. However, this would have allowed us to examine only sex and age, and not other predictors such as training, body mass index or history of injury. Accordingly, we developed a questionnaire that was implemented via the Internet to collect routinely available data from a large sample of recreational runners. Since our goal was to predict race performance, we used race velocity in meters per second (m/s) as the dependent variable. Independent variables were runner characteristics (sex, age and BMI) and training characteristics (typical training mileage and the use of sprint and tempo runs) that are known to be associated with race time.

### Subjects

The study questionnaire included items about age, sex, height and weight, training, injury, type of runner (on a 10 point scale from 1 - “endurance runner” - to 10 - “speed demon”), and type of running shoe (normal running shoe, minimalist, or Vibrams/sandals/barefoot). Questions on training specifically concerned the typical number of miles run per week and the maximum number of miles run in a single week leading up to the longest race, and whether the runner did interval training or tempo runs most weeks during training. Interval training involves short and intense periods at maximal effort followed by equal length or longer recovery periods of less strenuous exercise. Tempo runs are done at a steady pace at or slightly above the anaerobic threshold [13]. Participants were then asked to enter data for two or three recent races: distance and time plus subjective assessments of course difficulty (wind, hills, temperature) and fitness on race day.

The questionnaire was implemented via the Web on the news website *Slate.com* attached to a news story about race time prediction. Readers of an article criticizing the current approach to race time prediction were encouraged to “help Slate build a better predictor” and clicked on a link to the questionnaire. The text of the article made it completely clear that the deidentified data entered by participants would be used for data analysis by the study PI (AV). The project was discussed with the chair of the Memorial Sloan Kettering Cancer Center Institutional Review Board. Given that the project involved analysis of deidentified data and there was no potential for harm, it was deemed that no oversight was required. A copy of the questionnaire is included in the Additional file 1 (see “Questionnaire Text” on page 5- 8).

Use of the Internet to distribute a survey naturally raises questions as to two key aspects of survey validity: representativeness and selection bias. As regards representativeness, we planned to compare our sample with data on sex, age and race time from 50,266 participants in the 2013 New York Marathon and also with US national figures from Running USA [14]. We do not think that there is an important risk of selection bias in this study: there is no reason to believe that, say, a runner whose marathon time was faster than average given a certain 10 km time was any more or less likely to complete the questionnaire than a runner whose velocity slowed more dramatically from 10 km to marathon distance.

### Statistical analyses

Procedures for data inclusion, for instance, if a respondent included more than one race at a given distance, are given in the Additional file 1. We aimed to assess the association between race velocity (in m/s) and age, sex, BMI, and training (average mileage, intervals, tempo runs) [15, 16]. Since velocity varies by race distance, we created separate linear regression models for each variable of interest for each race distance. A change in velocity of the same magnitude has different implications based on initial velocity, so we explored modeling the logarithm of velocity, representing relative change in velocity. However, this model was found to explain less variation than using velocity untransformed. We tested for non-linearity in age, BMI and typical weekly mileage, and included cubic splines for these three covariates to account for non-linearity.

We used BMI as a correlate of body fat. Body fat likely affects race time directly – additional weight reduces run velocity over long distances – but is itself affected by training, with intense training leading to fat loss [17]. In addition, for individuals who are not overweight – about 80 % of the current sample – women tend to have lower BMI than men. Hence, we took a slightly different approach for examining the influence of BMI on race time. We modeled the association between velocity and age, velocity and BMI, and velocity and typical weekly mileage. The models were adjusted for age, typical weekly mileage, sex, intervals and tempo runs. Since BMI is highly correlated with weekly mileage and race training, the models for the association between velocity and age and between velocity and weekly mileage were calculated with and without BMI. Since the models with and without BMI produced comparable results, we included BMI in our descriptive models.

We also sought to develop a prediction model for race time. We first split the data 2:1 into a training (*n* = 1443) and validation set (*n* = 721), using a randomization algorithm that ensured a similar distribution of marathon runners and marathon times in each cohort. We then used the training cohort to explore multiple models for race time prediction using linear regression. The survey asked runners to tell us the difficulty of each race for which they reported a race time (very difficult, difficult, average, fast, or very fast). Very difficult or very fast races (5.6 % of the data) were not included in the data used to build the prediction models on the grounds that few runners reported race difficulty at the extremes. For difficult and fast races, we adjusted times to be more representative of a runner’s time for an “average” difficulty race of that distance. To do so, we created a model to predict race velocity in m/s for each race distance separately, adjusted for race difficulty (difficult, average or fast), sex, age, typical weekly mileage, intervals and tempo runs. The differences in velocity between difficult and average races, and between fast and average races, calculated in these models were used to adjust reported race times for difficult and fast races by adding the model coefficient to the runner’s true velocity, and then calculating a difficulty-adjusted time using the difficulty-adjusted velocity. The coefficients used to adjust for fast and difficult races for each race distance are reported in the Additional file 1 along with the prediction models.

A common formula for predicting race time for a longer race *y* based on a shorter race *x* was published by Riegel and is of the form: time for race *y* ÷ time for race *x* = (distance of race *y* ÷ distance of race *x*)^*k*^ [6]. The constant *k* represents the amount a runner’s velocity decreases as race distance increases (a “fatigue factor”). The models we tested included *k* as both a predictor, using a constant of *k* = 1.07, [6] and as the dependent variable, that is, using variables such as mileage to predict the correct value of *k*. Riegel cited a *k* of 1.08 for elite runners and 1.05 to 1.06 for male recreational runners aged 40 to 70 [6], while the Runner’s World online calculator uses a constant of 1.06 [1]. We chose a constant of 1.07 as an average, and performed sensitivity analyses using *k* = 1.06 and *k* = 1.08.

Models were compared using two metrics: mean squared error (MSE) and penalized mean squared error. Since overestimation of a runner’s velocity is more detrimental than underestimation – a runner who starts too slow can speed up during a race whereas a runner to starts too fast will slow dramatically - the penalized mean squared error was calculated by adapting the mean squared error formula, so that an overestimate of velocity had double the weight of an underestimate. All statistical analyses were two-sided, and significance was defined as *p* < 0.05. All analyses were conducted using Stata 13 (Stata Corp., College Station, TX). The full data set used in the analysis is provided as Additional file 2.

## Results

### Participant characteristics

The survey opened on April 24, 2014 and was closed for analysis on June 16, 2014. There were 2,497 responses. Runners were excluded if all races reported were the same distance (*n* = 171), if they were duplicates (*n* = 8), if they had only one unique race with full data (*n* = 7), as data from these participants cannot be used in a race time predictor. Participants were also excluded if they reported running a longer race in a shorter time than a race of a shorter distance (*n* = 6), or if the responses were obviously erroneous or falsified (*n* = 2). Erroneous data also lead to exclusion of race times from individual races reported by a participant (see Additional file 1 for details). The final cohort included 2,303 runners, with all runners having full data on at least two races of different distances. Only 3 men and 4 women reported a time that met Olympic qualifying standards for at least one race distance, hence our sample consists almost completely of recreational runners.

Characteristics of the study cohort are given in Table 1. Time and training data are given in Table 2. A detailed comparison of our cohort to US national data and the New York marathon is given in the Additional file 1. The average age and male:female ratio of our sample is similar. Although running velocities are faster on average, we did have good representation of slower runners, with over 300 runners in our cohort reporting marathon times longer than 4 h.

**Table 1:** Characteristics of study participants (*N* = 2,303)

Age	35 (29, 42)
Sex	
Female	890 (39 %)
Male	1,413 (61 %)
BMI	23.4 (21.7, 25.2)
Type of runner	
Strictly endurance	706 (31 %)
Generally endurance	1287 (56 %)
Generally speed	297 (13 %)
Strictly speed	13 (<1 %)
Typical weekly training mileage	30 (20, 42)
Any injury during training?	
Nothing that stopped me running	1564 (68 %)
Yes, I had to take a few days off	532 (23 %)
Yes, I had to take more than a week off from running	207 (9 %)
Ran intervals most weeks	1181 (51 %)
Did tempo runs most weeks	1328 (58 %)
Type of footwear	
Minimalist	465 (20 %)
Normal running shoe	1805 (78 %)
Vibrams, sandals, or barefoot	33 (1.4 %)

Given as median (IQR) or frequency (%). Data for all participants were available for all predictors listed in the table

**Table 2:** Age, sex, race training, velocity and time, by race distance

	5 km	5 mile	10 km	10 mile	Half-marathon	Marathon
	(*N* = 1,387)	(*N* = 313)	(*N* = 946)	(*N* = 357)	(*N* = 1,579)	(*N* = 1,022)
Age	34 (29, 42)	34 (28, 41)	35 (30, 43)	34 (29, 42)	35 (30, 43)	35 (30, 43)
Female	532 (38 %)	106 (34 %)	339 (36 %)	137 (38 %)	686 (43 %)	366 (36 %)
Typical Mileage	28 (18, 40)	25 (16, 40)	25 (18, 40)	28 (20, 40)	30 (20, 40)	40 (30, 50)
Intervals	716 (52 %)	165 (53 %)	462 (49 %)	177 (50 %)	839 (53 %)	579 (57 %)
Tempo Runs	773 (56 %)	164 (52 %)	535 (57 %)	208 (58 %)	942 (60 %)	684 (67 %)
Race Time Male	00:20:35 (00:18:20, 00:23:28)	00:34:59 (00:29:44, 00:41:04)	00:44:51 (00:39:48, 00:51:30)	01:14:21 (01:03:41, 01:23:08)	01:39:06 (01:28:00, 01:52:10)	03:28:02 (03:03:23, 03:57:36)
Race Time Female	00:26:01 (00:22:41, 00:29:09)	00:43:34 (00:37:32, 00:49:19)	00:54:58 (00:48:12, 01:02:08)	01:32:00 (01:20:11, 01:44:00)	01:56:32 (01:44:02, 02:12:16)	03:54:36 (03:31:40, 04:30:00)
Race Velocity Male	06:37 (05:54, 07:33)	07:00 (05:57, 08:13)	07:13 (06:24, 08:17)	07:26 (06:22, 08:19)	07:33 (06:43, 08:33)	07:56 (07:00, 09:04)
Race Velocity Female	08:22 (07:18, 09:23)	08:43 (07:30, 09:52)	08:51 (07:45, 10:00)	09:12 (08:01, 10:24)	08:53 (07:56, 10:05)	08:57 (08:04, 10:18)

Given as median (IQR) or frequency (%). Note that data from an individual runner will appear in two or three different columns. Race velocity is given as minutes per mile

### Characteristics associated with velocity

We first examined multivariable associations between runner characteristics and race time, both overall and separately by sex. We excluded data from the 5 and 10 mile distance due to the relatively limited number of runners at these distances. Sex, age, BMI, typical training mileage, interval training and tempo runs were all statistically significant predictors of race time at *p* < 0.0005. Results adjusted for BMI were very similar to those without BMI. Interaction analyses were used to determine whether the effect of these characteristics differed between males and females. Significant interactions were found between sex and age, typical mileage, BMI and interval training (*p* = 0.001, *p* = 0.004, *p* = 0.025 and *p* = 0.024, respectively), although differences were small. Men who ran intervals had a marathon time faster by 4:46 min on average, compared to 3:07 for females. For a one unit in BMI, increase in marathon time was about 40 s more for men. The difference in marathon time for training 50 vs. 30 miles per week was 25:32 for men and 31:41 for women; for age 50 vs. 30 the difference in time was 16:18 for men and 21:37 for women. If these associations are seen to be causal, then we could say that interval training has a greater effect on men, training volume has a greater effect on women, BMI has a greater effect on men and aging has a greater effect on women.

When typical training mileage was replaced by maximum training mileage in the models, results were similar, with maximum training mileage being significantly associated with race time for all race distances (*p* < 0.0001). However, maximum and typical weekly mileage were highly correlated (ρ > 0.9) and we decided to retain only typical weekly mileage for all analyses.

Fewer than 2 % of our cohort reported wearing Vibrams, sandals, or running barefoot. We excluded those runners from analyses of footwear. After adjustment for sex, age, BMI and training, runners wearing minimalist shoes had significantly faster race velocity than those reporting conventional footwear (*p* < 0.0001). Difference in race time was close to 0.5, 1.5, 2 and 3 min for 5 km, 10 km, half-marathon and full marathon respectively. There was no evidence that the association between footwear and velocity differed by race distance (*p* = 0.4 for interaction term).

**Fig. 1:**
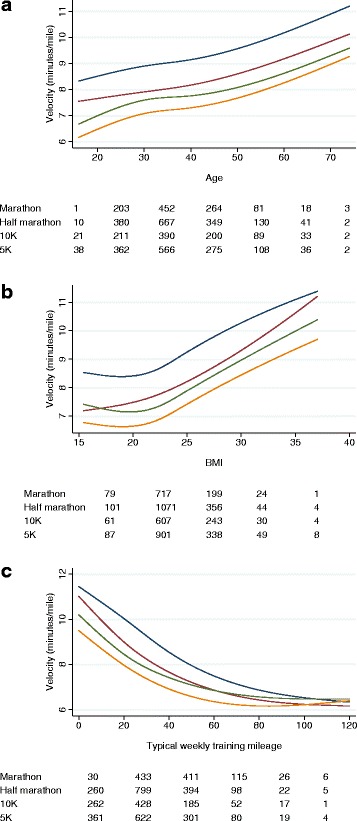
Race velocity in minutes per mile by age (**a**, adjusted for BMI and typical training mileage), BMI (**b**, adjusted for age and typical training mileage) and typical training mileage (**c**, adjusted for age and BMI). All models were also adjusted for sex and whether the runner trained with intervals or tempo runs. The tables underneath each figure represent the number of runners within the given age, BMI or mileage categories who ran a race of that distance. Yellow line: 5 km velocity; green line: 10 km velocity; red line: half-marathon velocity; blue line: marathon velocity

An additional question is whether the associations between race velocity and runner characteristics or training depend on race distance. For instance, we wanted to know whether the difference between male and female runners is greater for shorter compared to longer races. The results of these analyses are shown in Table 3 and Fig. 1a, b and c. Females do relatively better at longer distances, whereas tempo runs are associated with faster times more strongly for shorter distances. The association between interval training and race velocity was not found to differ by race length. Adjusting for BMI did not importantly influence these interactions. Differences between men and women were larger with adjustment for BMI, on the grounds that men are generally heavier; differences by intervals and tempo runs are smaller, suggesting that these types of training lower BMI. There was no significant interaction between race distance and age (*p* = 0.13) or between race distance and BMI (*p* = 0.4). Although there was a significant interaction between race distance and typical weekly mileage (*p* = 0.025), the magnitude was small, and the effect of typical weekly mileage on velocity did not differ importantly between race distances. These effects can be seen in Fig. 1a, b and c, where the curves for each race distance are approximately parallel.

**Table 3:** Multivariable analysis of race time

Covariate	Marathon	% change	Half Marathon	% change	10 km	% change	5 km	% change	Interaction *p*-value
Without adjustment for BMI									
Male	03:47:46		01:39:48		00:44:42		00:20:46		
Female	04:11:56	10.6 %	01:54:42	14.9 %	00:52:58	18.5 %	00:25:06	20.9 %	<0.0001
No tempo runs	04:02:55		01:48:17		00:49:59		00:23:15		
Tempo runs	03:52:50	−4.2 %	01:43:34	−4.4 %	00:46:22	−7.2 %	00:21:51	−6.0 %	0.002
No intervals	04:01:28		01:47:35		00:48:32		00:22:53		
Intervals	03:52:57	−3.5 %	01:43:39	−3.7 %	00:47:18	−2.5 %	00:22:01	−3.8 %	0.5
Adjusting for BMI									
Male	03:43:06		01:37:40		00:43:32		00:20:18		
Female	04:13:23	13.6 %	01:55:43	18.5 %	00:53:12	22.2 %	00:25:12	24.2 %	<0.0001
No tempo runs	03:59:38		01:46:52		00:49:05		00:22:49		
Tempo runs	03:51:15	−3.5 %	01:43:01	−3.6 %	00:45:56	−6.4 %	00:21:44	−4.7 %	0.005
No intervals	03:58:21		01:46:18		00:47:32		00:22:29		
Intervals	03:51:26	−2.9 %	01:43:04	−3.0 %	00:47:01	−1.1 %	00:21:55	−2.5 %	0.6

The model used here was adjusted for sex, intervals, tempo runs, age, and typical mileage, and separately with and without adjustment for BMI. After creating the model, all covariates except the covariate of interest were set to the mean, velocity was predicted and velocity converted to time in minutes

### Race time prediction

We found evidence that the Riegel formula predicted race time reasonably well for distances up to a half marathon (see Additional file 1: Figure S1 and S2), but was poor for marathon prediction. Using a linear regression model for each race distance with the intercept set to 0, we tested whether the coefficient for the Riegel race time predictions was equal to 1, which would indicate good calibration. The coefficient for Riegel marathon predictions was significantly different than 1 (*p* < 0.0001) and therefore poorly calibrated, while there was no evidence of a difference for half marathon and 10 k times, implying good calibration (*p* = 0.3 and *p* = 0.9, respectively).

We explored various models for marathon time prediction using the training set. We settled on two approaches. Model 1 was used for runners who provided data from only one prior non-marathon race. Model 1 predicted marathon velocity in m/s using typical weekly mileage and the predicted marathon velocity calculated using the Riegel formula where *k* = 1.07 and that runner’s longest non-marathon race. For runners who provided data from two prior non-marathon races, we calculated *k* between the two races provided. Model 2 used *k* between the two shorter races and typical weekly mileage to predict *k* between the longer reported race distance and a marathon. Variables strongly associated with velocity on univariate analysis were not found to improve prediction after controlling for training mileage and prior races times. Hence our models included only prior race time and typical weekly mileage.

Both velocity and *k* were then converted to time in minutes so that mean squared error and penalized mean squared error could be calculated. We also compared the error in these models to the predicted times using the Riegel model where *k* = 1.07 and the predictor time is from the runner’s longest reported non-marathon race. The formula for both models is given in the Additional file 1 (see “Formulae for Prediction Models” on pages 3 – 4).

**Fig. 2:**
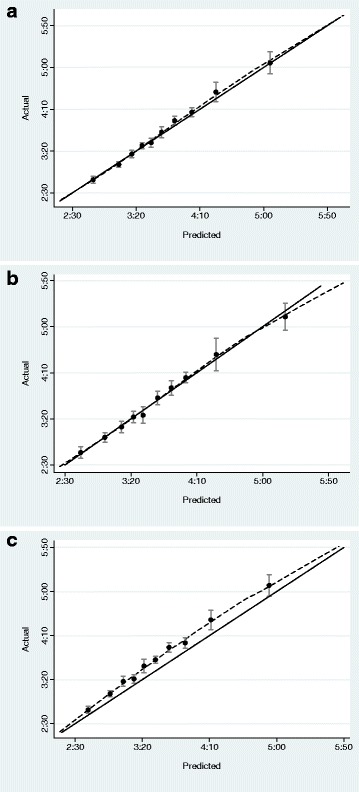
Calibration plots comparing observed marathon times to those predicted by Model 1 (**a**, using information from one prior race), Model 2 (**b**, using information from two prior races), and the Riegel formula (**c**, where *k* = 1.07 and the shorter race is the longest reported non-marathon race)

All mean squared errors were calculated on the same sample of runners in the validation data (*N* = 156) who reported running a marathon and two other races of differing non-marathon distances. Mean squared errors for model 1, 2 and Riegel were 227.6, 208.3 and 380.7, respectively. MSEs penalizing underestimation of race time were 646.1, 525.0 and 1429.8. Figure 2a, b and c show calibration plots. Calibration of the novel models (Fig. 2a and b) is excellent; in contrast, the Riegel formula (Fig. 2c) shows clear miscalibration with actual running times considerably slower than those predicted. Table 4 shows the distribution of differences between predicted and observed times. Predicted marathon times from Riegel are 10 min or more too fast for about half of all runners; for the new models, this drops to about 25 % of runners. Sensitivity analyses using different coefficients did not change our conclusion. Using the Riegel formula with *k* = 1.08 did improve estimates slightly, but MSEs were much higher than those from the novel models with marathon velocity overestimated for about 75 % of runners.

**Table 4:** Distribution of residuals

Centile	Model 1	Model 2	Riegel
5^th^	−23:53	−21:38	−36:17
10^th^	−18:25	−16:47	−30:24
25^th^	−9:55	−7:31	−19:47
33^rd^	−7:18	−4:53	−16:50
50^th^	−1:47	0:23	−10:09
67^th^	3:21	4:46	−5:08
75^th^	6:28	7:20	−2:48
90^th^	11:49	15:01	2:53
95^th^	15:23	21:05	5:12

The table shows differences between predicted and observed race times. A negative time indicates that predicted race time is shorter than observed, that is, predicted velocity is too fast. The table shows that, for instance, nearly 25 % of runners have a predicted race time from the Riegel formula greater than 20 min too fast

## Discussion

We obtained data on over 2000 recreational runners using an Internet based survey. Using this unique data set, we studied predictors of endurance running performance in terms of runner characteristics and training. We also built a novel statistical model to predict marathon time based on race performance at shorter distances. Our study is distinguished by its size and its focus on recreational rather than elite athletes. In addition, we avoided predictors such as VO_2_max that require laboratory measurement, and only included predictors, such as typical weekly mileage, that would be readily known by any runner.

Some of our findings concerning training and race time go somewhat against conventional wisdom. For instance, it is typically believed that training volume is more important for distances such as the marathon than for the 5 and 10 km (km) distance [18–20]. In contrast, we found that the association between training mileage and race velocity is similar across race distances. Similarly, interval training is thought to be of most benefit for shorter distances, with tempo runs seen to be of particular value for long races: typical training plans include more frequent interval training, but less emphasis on tempos, for 10 km races than for marathons [21–23]. We found that tempo runs were more strongly associated with velocity for short distances and that interval training had a similar association with velocity irrespective of distance.

The conventional wisdom that women do relatively better as race distance increases [24] was supported by our findings: women were about 20 % slower than men for the 5 km distance; this difference dropped to 10 % for the marathon. On the other hand, the conventional wisdom that race velocity for longer distances is less affected by age than for short distances was not supported, as reductions in velocity with age were similar across distances.

Our other major finding was that although standard race prediction tools based on the Riegel formula work well for distances up to a half marathon, they substantially underestimate time for the marathon. Given the importance of pacing for marathon distance, this finding has considerable implications. Our novel marathon prediction model is straightforward and could easily be implemented on any website. A version of our model which uses a more simple adjustment for race difficulty is currently available at *Slate.com* [25]. The model presented in this paper has been slightly updated using a more specific adjustment for race difficulty.

Given the observational nature of the data, it is worth reflecting on whether it is reasonable to make causal attributions between training and velocity. We believe that it is in fact justifiable to draw conclusions such as that a runner who incorporates interval training should expect about a 3 % decrease in race time (Table 3). Not only do our findings have biologic plausibility – there is an extensive literature on the biology of interval training [5] – and an appropriate dose-response gradient (such as that shown in Fig. 1c), but alternative explanations for our findings would appear to be unconvincing. In theory, runners whose training includes, say, tempo runs, might be more likely to have muscular or metabolic factors that increase velocity, but such an effect would seem unlikely, and there is certainly no direct evidence for it.

There are two limitations of our study. First, although the sample size is up to 200 times larger than some previous studies, numbers are limited in some subgroups. For instance, only 21 of our marathon runners are aged over 60 and only 3 are aged over 70. This limits our ability to make confident predictions in these age groups. Second, concerns may be raised over the representativeness of our sample. As argued above, we have no reason to believe that use of the Internet to obtain data would lead to selection bias for the questions of interest in this study. For instance, the relationship between interval training and race velocity is highly unlikely to differ by Internet access or propensity to complete Internet surveys. Our cohort was younger and faster than participants in the New York marathon, but reasonable representation of slower and older runners, and the use of modeling techniques that are based on the full data set, limit the influence of this aspect of our study.

It might be argued that the use of participant self-report is a weakness of our approach. However, the objective of this study was to use information easily available to the recreational runner. Further, it is unclear that results would have been importantly affected had alternative sources of information been used. Take, for instance, tempo runs during training. The alternative to self-report would be to have had a running coach visit participants, watch a tempo run and verify a running log to determine whether the participant ran a tempo most weeks during training. Not only would such an approach be of highly doubtful feasibility, but there are no obvious reasons to doubt that trainer evaluation and participant self-report would be markedly different. Self-report of tempo runs – indeed, all aspects of runners and training we recorded – were associated with race time in the anticipated direction. This lends support to our study methodology.

We note that were a single big city marathon to conduct a similar Internet-based survey as part of entry requirements, a sample size perhaps 10 times larger than ours could be achieved, and there could be little question about representativeness. The questionnaire we used is relatively short, and so questionnaire completion would not incur an undue burden on participants. We encourage others to repeat our study using such an approach.

## Conclusions

We obtained data on a large number of recreational runners in order to develop predictors of endurance race time. Our findings can be used to make fair comparisons between runners of different ages and sexes, inform training regimens and make better prediction of race times, allowing better pacing.

## Additional files

Additional file 1: BMC Supplementary materials. **Figure S1.** Calibration plot comparing times predicted using Riegel formula where k = 1.07 and the shorter race is the longest reported non-half-marathon race to observed half-marathon times. **Figure S2.** Calibration plot comparing times predicted using Riegel formula where k = 1.07 and the shorter race is the longest report non-10Km race to observed 10 K times. (DOCX 33 kb)

Additional file 2: Master Data Predicting Races Times Final. (XLSX 377 kb)
