# Supplementary material for: An empirical study of race times in recreational endurance runners
Source: BMC Sports Sci Med Rehabil. 2016 Aug 26;8(1):26. doi: 10.1186/s13102-016-0052-y (PMC5000509; doi:10.1186/s13102-016-0052-y)
Supplement: Supplementary file 1 — BMC Supplementary materials. Figure S1. Calibration plot comparing times predicted using Riegel formula where k = 1.07 and the shorter race is the longest reported non-half-marathon race to observed half-marathon times. Figure S2. Calibration plot comparing times predicted using Riegel formula where k = 1.07 and the shorter race is the longest report non-10Km race to observed 10 K times. (DOCX 33 kb) [file 13102_2016_52_MOESM1_ESM.docx]

**Supplementary Materials**

**Procedures for data inclusion**

Race data were excluded if there were missing data on race distance, time, difficulty, or fitness level at race time, or if velocity was < 4 or > 20 minutes per mile. If a respondent reported two races of the same distance, only one race was included in the analysis. For races of the same distance with the same race difficulty and fitness level at race time, the average of the two race times was used. If race difficulty was reported as “very fast” or “very difficult” for one race and “fast”, “difficult” or “average” for the other race, the “very difficult” or “very fast” race was excluded. If fitness level for one race was reported as “I’d trained some” or “not well prepared” and the fitness level for the other race was reported as “in good shape” or “best shape of my life”, then the race with the lower level of physical fitness was dropped. For any remaining duplicate race distances, one of the two races was selected randomly for inclusion.

**Comparison of our cohort with normative data**

In our cohort, 36% of participants were female, similar to 39% of participants in the 2013 New York Marathon and 43% of marathon participants reported by Running USA. Comparing the age distribution to the New York Marathon, which had a median age of 38 for females and 42 for males, we found that our cohort was slightly younger, with a median age of 33 for females and 37 for males. Overall, our cohort had median marathon times about forty minutes faster than the New York Marathon and those reported by Running USA. The median marathon time for females in our cohort was 3:54:36, compared to median female time in the New York Marathon (4:38:01) or reported by Running USA (4:41:38). For males, our median time was 3:28:02, compared to 4:11:17 in the New York Marathon and 4:16:24 reported by Running USA. Nonetheless, we did have a reasonable number of slower runners. Out of 890 female runners in our sample, 73 had marathon times between 4:00 and 4:30, 55 had times between 4:30 and 5:00, 31 had times between 5:00 and 6:00 and 6 had times greater than 6:00. Out of 1,413 males, 88 had marathon times between 4:00 and 4:30, 31 had times between 4:30 and 5:00, 24 had times between 5:00 and 6:00 and 7 reported marathon times longer than 6:00. Overall, 19% of female marathon runners and 11% of male marathon runners in our sample reported marathon times longer than 4 hours.

**Calibration plots for Riegel formula in race time prediction for half-marathon and 10km**

**Figure S1.** Calibration plot comparing times predicted using Riegel formula where k=1.07 and the shorter race is the longest reported non-half-marathon race to observed half-marathon times.

**Figure S2.** Calibration plot comparing times predicted using Riegel formula where k=1.07 and the shorter race is the longest report non-10Km race to observed 10K times.

**Formulae for Prediction Models**

All distances are entered into the equations as meters and all times are entered as seconds. The final model will give marathon time in minutes. Races that were reported by the runner to be “very difficult” or “very fast” were excluded. Typical mileage is the number of miles run in a typical training week.

For each race, first adjust the race time if race was reported as “difficult” or “fast”.

If race difficulty was “average”, ${adj\_time}_{r}= {time}_{r}$

If race difficulty was “difficult” or “fast”, ${adj\_time}_{r}=\frac{{distance}_{r}}{(\frac{{distance}_{r}}{{time}_{r}}+ adj)}$

Velocity adjustment factors, by race distance:

| Race Distance | Difficult Race (adj) | Fast Race (adj) |
| --- | --- | --- |
| Half-marathon | 0.0335971859175381 | -0.0978322644420439 |
| 10 mile | 0.1030755530328555 | -0.1358099643292151 |
| 10K | 0.024557694615445 | -0.0780677777771365 |
| 5 mile | 0.1089566001045939 | -0.1549942921949754 |
| 5K | 0.1129432382020499 | -0.0237814322487082 |

**Model 1**

Model 1 is for runners who reported only one non-marathon race (*r1*).

$${velocity}_{Riegel}=\frac{42195}{(adj\_time_{r1})\times\left( \frac{42195}{distance_{r1}} \right)^{1.07}}$$

Using${velocity}_{Riegel}$, calculate predicted velocity $({velocity}_{Model1})$:

$${velocity}_{Model1}=0.16018617+\left( 0.83076202\times{velocity}_{Riegel} \right)+ (0.06423826\times\frac{typical mileage}{10})$$

$$tim{e (in minutes)}_{Model1}=\frac{(42195 \div60)}{{velocity}_{Model1}}$$

**Model 2**

Model 2 is for runners who reported two non-marathon races (shorter race *r1* and longer race *r2*).

$$k_{\frac{r2}{r1}}=\frac{\ln\left( \frac{adj\_time_{r2}}{adj\_time_{r1}} \right)}{\ln\left( \frac{distance_{r2}}{distance_{r1}} \right)}$$

$$predicted k_{marathon}=1.4510756+\left( -0.23797948\times k_{\frac{r2}{r1}} \right)+(-0.01410023\times\frac{typical mileage}{10})$$

$$predicted marathon time in minutes=\frac{\left( (adj\_time_{r2})\times\left( \frac{42195}{distance_{r2}} \right)^{predicted k_{marathon}} \right)}{60}$$

**Questionnaire Text**

**Help us build a better running calculator**

We are creating a tool to help runners accurately calculate their predicted finish times for races. To do this, we’re collecting real-life data from runners. You can help us by completing this brief survey, which takes about five minutes. We’ll ask a few basic questions about you, and you’ll need to provide finish times from at least two races of DIFFERENT lengths. Ideally, you’ll have run these races within a six-month period, but it is fine to include races that are more than six months apart so long as you were around the same age and were at roughly the same fitness level.

**Your age**

**Sex** (Male/female)

**Height: Feet**

**Height: Inches**

**Weight (lbs)**

**Are you an endurance runner or a speed demon?**

Endurance runner 1 2 3 4 5 6 7 8 9 10 Speed Demon

**What type of footwear do you wear?**

Normal running shoe

Minimalist

Vibrams, sandals, or barefoot

**Now tell us about some recent races.**

Please input only recent races, ideally ones that you have run within the last six months. Don't include a race unless you were trying as hard as you can (as opposed to, say, keeping a friend company at a slower pace).

**Race 1**

**Distance**

5K / 5 miles / 10K / 10 miles / Half-marathon / Marathon

**Your time**

HH:MM:SS

**How difficult was the course?**

Very difficult: Very hilly, hot, or windy

Difficult: Hilly, hot, or windy

Average

Fast: Cool, calm, and flat

Very fast: Downhill or tailwind

**How do you rate your fitness at the time of the race?**

As fit as I’ve ever been

Good shape, but I could have trained a bit harder

I’d trained some

I wasn’t well prepared

**Race 2**

Please enter in a race that was a different distance than Race 1.

**Distance**

5K / 5 miles / 10K / 10 miles / Half-marathon / Marathon

**Your time**

HH:MM:SS

**How difficult was the course?**

Very difficult: Very hilly, hot, or windy

Difficult: Hilly, hot, or windy

Average

Fast: Cool, calm, and flat

Very fast: Downhill or tailwind

**How do you rate your fitness at the time of the race?**

As fit as I’ve ever been

Good shape, but I could have trained a bit harder

I’d trained some

I wasn’t well prepared

**Race 3 (optional)**

Please enter a race that was a different distance than Race 1 and Race 2.

**Distance**

5K / 5 miles / 10K / 10 miles / Half-marathon / Marathon

**Your time**

HH:MM:SS

**How difficult was the course?**

Very difficult: Very hilly, hot, or windy

Difficult: Hilly, hot, or windy

Average

Fast: Cool, calm, and flat

Very fast: Downhill or tailwind

**How do you rate your fitness at the time of the race?**

As fit as I’ve ever been

Good shape, but I could have trained a bit harder

I’d trained some

I wasn’t well prepared

**Now tell us about your training for the longest race that you gave us a time for.**

**What was your typical weekly mileage leading up to this race?**

**What was the maximum number of miles you ran in a single week during training?**

**Did you run sprints, intervals, or hill repeats most weeks during training?**

Yes

No

**Did you do tempo runs most weeks during training? (If you don’t know what a tempo run is, you probably didn’t run one!)**

Yes

No

**Did you have an injury?**

Nothing that stopped me running

Yes, I had to take a few days off

Yes, I had to take more than a week off from running
